# Breaking Boundaries in the Brain—Advances in Editing Tools for Neurogenetic Disorders

**DOI:** 10.3389/fgeed.2021.623519

**Published:** 2021-02-01

**Authors:** Bronte A. Coorey, Wendy A. Gold

**Affiliations:** ^1^School of Medical Sciences, The University of Sydney, Sydney, NSW, Australia; ^2^Molecular Neurobiology Research Laboratory, Kid's Research, Children's Hospital at Westmead, Westmead, NSW, Australia; ^3^Kids Neuroscience Centre, Kids Research, Children's Hospital at Westmead, Westmead, NSW, Australia; ^4^Discipline of Child and Adolescent Health, Faculty of Medicine and Health, Camperdown, NSW, Australia; ^5^Molecular Neurobiology, The Children's Medical Research Institute, Westmead, NSW, Australia

**Keywords:** gene therapy, neurogenetic, CNS, gene editing, mutations

## Abstract

Monogenic neurological disorders are devastating, affecting hundreds of millions of people globally and present a substantial burden to individuals, carers, and healthcare systems. These disorders are predominantly caused by inherited or *de novo* variants that result in impairments to nervous system development, neurodegeneration, or impaired neuronal function. No cure exists for these disorders with many being refractory to medication. However, since monogenic neurological disorders have a single causal factor, they are also excellent targets for innovative, therapies such as gene therapy. Despite this promise, gene transfer therapies are limited in that they are only suitable for neurogenetic disorders that fit within the technological reach of these therapies. The limitations include the size of the coding region of the gene, the regulatory control of expression (dosage sensitivity), the mode of expression (e.g., dominant negative) and access to target cells. Gene editing therapies are an alternative strategy to gene transfer therapy as they have the potential of overcoming some of these hurdles, enabling the retention of physiological expression of the gene and offers precision medicine-based therapies where individual variants can be repaired. This review focusses on the existing gene editing technologies for neurogenetic disorders and how these propose to overcome the challenges common to neurogenetic disorders with gene transfer therapies as well as their own challenges.

## Introduction

Since the first proof-of-concept human gene therapy in 1990, the field of gene and cell therapies has made significant advances resulting in almost 3,000 clinical trials being tested on a broad range of genetic disorders. Neurogenetic disorders including Fragile X syndrome, Rett syndrome, and Huntington's disease, encompass disorders of the central and peripheral nervous systems and pose severe difficulties for individuals who consequently experience debilitating lifestyles. Symptoms common to many neurogenetic disorders include seizures and cognitive and physical disabilities, which severely affect the individual's quality of life.

Many neurogenetic disorders are monogenic Mendelian disorders, caused by variants in genes required for normal function of the brain, spinal cord, peripheral nerves, and muscles. Despite the headway in gene replacement therapies such as for Spinal muscular atrophy (Mendell et al., 2017), developing gene therapies for Mendelian monogenic neurological disorders remains challenged due to the complexity of the brain and the central nervous system, the limited regenerative capacity of neurons, and the impenetrable blood–brain barrier. Neurons, the major cells of the nervous tissue, are morphologically and physiologically heterogeneous, are non-dividing and are strictly organized to form complex circuits making them challenging cells to target.

Neurogenetic disorders are a very heterogenous group of disorders with different inheritance patterns (recessive, dominant, and dominant negative) and mechanism of action [gain of function (GOF) and loss of function (LOF)] characteristics sometimes observed in individuals within the same disorder, making blanket gene therapies extremely challenging. In addition, the expression of some genes are highly dose sensitive.

In recent years, research into neurogenetic disorders has made remarkable advances owing to the advent of genotyping arrays and next-generation sequencing such as whole genome and exome sequencing. These improvements have led to the identification of novel rare disease-causing variants and facilitated a greater understanding of the etiology of many neurological disorders. Many of these neurogenetic disorders are minimally responsive and refractory to conventional therapies, highlighting the critical need for alternate approaches to patient care such as gene therapies (Gribkoff and Kaczmarek, 2017; Joshi et al., 2017).

Gene and cell therapies encompass a suite of therapies that pose to alter the expression of a disease-causing gene and are constantly evolving with new therapies such as gene transfer therapy (e.g., Spinal Muscular Atrophy), gene editing [e.g., Leber Congenital Amaurosis 10 (LCA10)], CAR-T cell therapies (e.g., Acute Lymphoblastic Leukemia) and improved delivery mechanisms such as recombinant adeno associated viral (rAAV) and lentiviral vectors, being tested in, and emerging from, clinical trials. Gene editing tools provide a much-needed translational pathway for neurogenetic disorders as they strive to overcome the many challenges faced by these disorders at the gene level such as dosage sensitivity and dominant-negative expression, and at the cellular level where the target cells (neurons) are non-dividing.

## Gene Transfer Therapy

Gene transfer therapies, where synthetic transgenes are introduced into cells to compensate for the lack of expression of a faulty gene, are currently being tested in clinical trials for many neurogenetic disorders. Although, proving to be successful for some neurogenetic disorders, gene transfer therapies are not without their challenges which include: the correct delivery modality (e.g., rAAV or lentival delivery) such that the target cell population receives adequate copies of the introduced gene, immune rejection where patients may have neutralizing antibodies against the introduced vector, or may elicit an overactive immune response to the foreign cDNA, ethical issues surrounding germline modifications, route of delivery, and the high cost of therapy. rAAV vectors are widely used as a delivery modality in clinical trials for the *in vivo* delivery of synthetic transgenes. AAVs have demonstrated low-pathogenicity, replication incompetency, broad-tropism to cell types, and ease of manipulation (Wang et al., 2019). Eleven naturally occurring and several synthetic serotypes, for example LK03 exist which are differentiated by their different capsid proteins that govern their tropism (Lisowski et al., 2014). This leads to cell specific tropism for example AAV9 which, unlike other natural occurring AAVs, is able to cross the blood brain barrier and target cells of the brain such as neurons, astrocytes and microglia. Furthermore, to broaden the scope of capsid tropism and overcome neutralizing antibodies the production of, synthetic capsids has increased exponentially (Westhaus et al., 2020).

Since the first approved gene therapy clinical trial in 1990, almost 3,000 trials have (and are) being tested to treat an array of diseases and disorders by providing a synthetic copy of the defective gene to restore functional expression (Blaese and Anderson, 1990; Blaese et al., 1995). Of the current gene therapies, rAAV mediated gene transfer therapies, particularly rAAV9, are the most adopted delivery method for neurological disorders due to their cell-specific transduction capabilities. rAAVs are favorable for CNS delivery as they are small and can transduce both dividing and non-dividing cells. Of all the gene transfer therapies currently in clinical trials, many utilize rAAVs due to their specific cellular tropism. For example, AAVrh.10 and AAV2 are used for intraparenchymal delivery and AAV9 for systemic and intrathecal delivery. Different delivery routes have, and are being tested in gene therapy clinical trials of neurogenetic disorders with systemic delivery showing success for Spinal Muscular Atrophy (Al-Zaidy et al., 2019), intrathecal delivery for Batten Disease (Cain et al., 2019) and direct injection into specific regions of the brain for Sanfilippo type B syndrome via intra-cerebral administration (Tardieu et al., 2017).

A variety of delivery modalities and delivery routes exist alongside AAV-mediated systemic delivery for neurogenetic disorders. Virus mediated gene transfer therapies are most commonly used due to their transduction efficiency and include rAAVs, adenovirus vectors, retrovirus, and lentivirus vectors. Alternatively, non-viral methods of delivery are also being investigated such as nanotechnology that packages and encapsulates the therapy in nanoparticles composed of biomaterials such as phospholipids and polymers.

Gene transfer therapy, can be driven by a tissue specific promoter (e.g., synapsin), a generic promoter (e.g., CMV) or a minimal endogenous promoter. Despite these, the expression of the transgene is not regulated by the natural regulatory elements at the gene's native locus. This limitation impacts many genetic neurodevelopmental disorders which fall into the “Goldilocks” area of expression where the unregulated overexpression of the transgene also exacerbates disease. A perfect example is the *MECP2* gene, where insufficient levels result in Rett syndrome and overexpression results in MECP2-duplication syndrome (Amir et al., 1999; Van Esch, 2012). Thus, there is a clear imperative to develop therapies for these disorders that reinstate the *natural* expression and regulation of the defective gene.

## Gene Editing Technologies

The capabilities of gene transfer therapy falls short for many neurogenetic disorders that are either dose dependant (where the unregulated overexpression of the synthetic gene is also detrimental), dominant-negative (where the gene product of the mutant allele adversely affects the wild type allele), or where the gene is just too large to fit into an rAAV vector (which has a packaging capacity of 4.7 kB). A clear imperative to develop gene therapy strategies for these disorders has resulted in the emergence of gene editing technologies that offer to correct mutations and retain endogenous expression of the gene of interest.

There are three predominant editing technologies that enable the manipulation of cellular DNA at the native locus. These are zinc finger nucleotides (ZFNs), transcription activator like effector nucleases (TALENs), and clustered regularly interspaced short palindromic repeats (CRISPR) and CRISPR associated (Cas) proteins.

The first reported programmable nucleases which could target specific DNA sites for cleavage were ZFNs (Kim and Chandrasegaran, 1994; Kim et al., 1996). ZFNs create a site-specific cleavage in DNA with an adapted FokI endonuclease that enables DNA alteration in the repair process of the introduced double stranded break (DSB). Building on this strategy, a more versatile system coined TALENs was created that combined the FokI endonuclease with transcription activator-like effectors (TALEs) modular DNA binding domains (Carlson et al., 2012). TALENs are able to recognize unique, randomly selected target sequences, and can be engineered for targeted gene modifications (Christian et al., 2010). The introduction of the CRISPR/Cas system in 1987 transformed the gene editing field in that it offers an easy to use, malleable tool that generates precise programmable cuts in genomic DNA (Barrangou et al., 2007). The associated Cas enzyme can be guided by a single guide RNA to precise recognition sites located near protospacer adjacent motif (PAM) sites where DSB are introduced (Barrangou et al., 2007). Various bacteria exist with an array of Cas enzymes that can be manipulated to suit the appropriate need such as *Streptococcus pyogenes* Cas9 (SpCas9) and *Staphylococcus aureus* Cas9 (SaCas9) which differ by their PAM sites as well as the enzyme size. SaCas9 (3156 bp) is significantly smaller in size than SpCas9 (4101 bp) and requires a more specific PAM region of sequence NNGRRT as opposed to the more adaptable NGG sequence associated with SpCas9. Other Cas enzymes such as Cas9 and Cas12 create blunt end and staggered DSBs, respectively, enhancing the tool-kit of Cas-mediated gene editing therapies (Banakar et al., 2020).

Gene editing strategies involve the introduction of a break in the genome at a specific allocation by a nuclease enzyme, which takes advantage of the cells natural repair mechanisms to fix the damage, and provides a window of opportunity for editing to occur. Genomic editing events rely on one of two of the cell's natural repair pathways, homology directed repair (HDR), and non-homologous end joining (NHEJ). HDR occurs during all phases of the cell cycle and although it is more common in dividing cells, is highly inefficient in non-dividing cells. Alternatively, NHEJ which is commonly used in non-dividing cells such as neurons, is more efficient and error prone in all cell types (Iyama and Wilson, 2013). Taking advantage of the NHEJ repair pathway, the research field has developed tools to edit post mitotic cells such as neurons, thus enabling gene editing strategies. To date, there are six main novel gene editing strategies for non-dividing cells which have been developed and allow DNA editing of post mitotic cells. These strategies include PITCh (precise integration into targeted chromosome) (Nakade et al., 2014), HITI (homology independent targeted integration) (Suzuki et al., 2016), vSLENDR (virus-mediated single-cell labeling of endogenous proteins via HDR) (Nishiyama et al., 2017), HMEJ (homology mediated end joining) (Yao et al., 2017), CRISPR Prime editing (Anzalone et al., 2019), and the HITI based SATI approach (Single homology Arm donor mediated intron-Targeting Integration) (Suzuki et al., 2019).

Many gene editing clinical trials are underway testing *ex vivo* approaches whereby patient cells are collected, edited outside of the patient and the modified cells are then transplanted back into the patient to exert a therapeutic effect. However, currently, the only *in vivo* gene editing therapy being tested in clinical trials is LCA-10, which is designed to correct pathogenic variants in the *CEP290* gene in individuals with Leber congenital amerosis (ClinicalTrials.gov Identifier: NCT03872479). An appealing and significant feature of the LCA-10 trial is the localization of delivery of the therapy where the CRISPR editing technology is delivered directly to the target site through injection in the eye, eliminating any potential off-target effects in other cells and organs. To date 38 CRISPR/Cas editing, 13 ZFN and 6 TALENs-mediated gene editing strategies are currently under investigation or have been tested in clinical trials. Of these trials, there are no studies that have, or are testing editing *ex vivo* technologies in neurological disorders.

## Discussion

### Overcoming the Regulation of Gene Expression in Neurological Disorders

One of the main concerns of gene transfer therapies is controlling gene regulation where the overexpression of the introduced gene is either ineffective or equally as detrimental. Each gene has it's own nuances which dictate whether therapies will be appropriate or not. Dosage dependent genes such as *MECP2*, where mutations cause Rett syndrome (Amir et al., 1999), and the dominant-negative expression of the *FBN1* gene in Marfan syndrome (Robinson and Godfrey, 2000) are prime examples where gene transfer therapies are ambitious. Gene transfer therapy lacks the precision required to maintain the exogenous expression levels of the gene due to uncontrolled viral transduction efficiency resulting in many (up to hundreds) of gene copies per cell. For example, *MECP2* gene expression in Rett syndrome is tightly regulated by endogenous regulatory elements and studies have demonstrated that over-expression is as detrimental and under-expression of the gene (Van Esch, 2012). Therefore, *MECP2* requires endogenous regulation of dosage for effective therapeutic benefit. Additionally, gene transfer therapy is unable to compensate for the effects of dominant negative alleles. Dominant negative mutations arise when the mutant allele interferes with the function of the remaining wild-type allele, causing a >50% loss of function. For example, a study identified that a p.T258M mutation in the KIF1A gene exerts a dominant-negative effect on the wild type KIF1A, whereby this mutation actually suppresses all KIF1A activity (Cheon et al., 2017). Individuals with these variants, are not likely to benefit from gene transfer therapy as this therapy will not alleviate the detrimental effects of the mutant allele in the cell (Glorioso et al., 2015).

### Challenges of Gene Editing

Alongside the promising benefits that gene editing holds, careful consideration of the long term and short-term consequences and downstream effects need to be considered. Despite CRISPR/Cas9 gaining increased therapeutic interest, its application has been under controversial scrutiny lately where CRISPR/Cas9 was used to permanently alter the genomes of unborn children in the hope of making them immune from HIV infection (Lovell-Badge, 2019). Additionally, and importantly, this has also led to critically important discussions regarding the ethics of human genome editing, in particular, inherited changes to the human genome that can be passed down to future generations.

The long-term implications of CRISPR/Cas9 will only be completely understood when the current human clinical trials are completed, and the treated individuals are monitored over time. Although some studies have observed large deletions and rearrangements at various sites along the genome in mitotically active cells (Kosicki et al., 2018), this effect has not yet been identified in post-mitotic neurons and may not be applicable to neurogenetic disorders as it could be specific to mitotic replication cycles only. Another concern is cell-mediated immune responses to the CRISPR/Cas9 machinery (Crudele and Chamberlain, 2018; Charlesworth et al., 2019). Approximately 40% of the human population is colonized with naturally occurring *S. aureus* and 20% with S. *pyogenes*, with a large percentage having antibodies and T cells against both bacteria (Roberts et al., 2012). Knowing that pre-existing humoral and cell-mediated adaptive immune responses to Cas9 exists in humans, care should be taken as the CRISPR/Cas9 system moves toward clinical trials. Moreover, rAAV vector integration at CRISPR-induced DNA break sites has recently been reported in pre-clinical studies (Hanlon et al., 2019). Despite sequences of naturally occurring rAAVs found commonly to be integrated into the human genome at ~47%, the consequences of integrated sequences from a synthetic AAV strain remains unclear (Hanlon et al., 2019).

CRISPR/Cas9-mediated cancer concerns have also been noted (Schaefer et al., 2017). Neurons have a very active response to DNA damage where even just one or two CRISPR-Cas9 induced DNA cuts can lead to toxicity resulting in cell death. To ensure the survival of the cell, introduction of double stranded breaks in DNA initiates the cellular repair strategies NHEJ or HDR. Double stranded breaks may also induce a p53 mediated DNA damage response or lead to mutations in the p53 gene, which plays an active role in regulating cell division and death, resulting in rapid growth of unwanted cell populations (Yla-Herttuala, 2018). Despite the claims by Schaefer et al. being subsequently retracted, (Nutter et al., 2018) these preliminary findings raise important questions on how CRISPR-induced p53 dysregulation may result in abnormal and uncontrollable cell division, and an increased cancer risk. Additionally, the unfettered CRISPR/Cas9 cutting capacity is of concern. However, mechanisms to mitigate this are being developed such as phage derived anti-CRISPRs that act as a CRISPR “kill switch” to inactivate the Cas9 enzyme from further cutting (Bondy-Denomy et al., 2013). The anti-CRISPRs can be delivered in conjunction with the Cas9 gene editing therapy which halts gene editing activity and may also possibly limit off-target effects (Shin et al., 2017). Further studies beyond yeast experiments will elucidate this promising control mechanism (Basgall et al., 2018).

The consequences of gene editing and the effect that editing has on gametes and heritability in further generations is not well-understood and needs to be considered prior to clinical applications (Niemiec and Howard, 2020). Although aiming to edit only somatic cell lines, off-targeted effects could possibly result in editing reproductive cells and have implications on the gametes which can be passed on to further generations (Niemiec and Howard, 2020).

## Conclusion

Gene therapy has grown significantly over the past three decades and is continuing to flourish and provide promise for the many individuals affected by incurable neurogenetic disorders. Gene transfer therapies are trailblazing the translational path, progressing from pre-clinical studies to clinical trials. Gene editing therapies are following closely, with the potential to provide novel therapies for millions of individuals with neurogenetic disorders as they overcome the hurdles encountered by conventional gene transfer therapies. Increased investment into gene editing pre-clinical studies as well as the associated governance and ethical standings is required to bring the benefits to those mostly affected.

## Author Contributions

BC and WG equally contributed to the writing of the manuscript, the editing of the manuscript, and the concepts included in the manuscript.

## Conflict of Interest

The authors declare that the research was conducted in the absence of any commercial or financial relationships that could be construed as a potential conflict of interest.
